# Supplementary material for: Adiabatic mode transformation in width-graded nano-gratings enabling multiwavelength light localization
Source: Sci Rep. 2021 Jan 12;11:669. doi: 10.1038/s41598-020-79815-9 (PMC7804207; doi:10.1038/s41598-020-79815-9)
Supplement: Supplementary file 1 — Supplementary Information 1. [file 41598_2020_79815_MOESM1_ESM.docx]

**Adiabatic Mode Transformation in Width-graded Nano-gratings Enabling Multiwavelength Light Localization**

Moein Shayegannia^1^, Arthur O Montazeri ^1,†^, Katelyn Dixon^1^, Rajiv Prinja^1^, Nastaran Kazemi-Zanjani^1^ and Nazir P Kherani^1,2,*^

^1^Department of Electrical and Computer Engineering, University of Toronto, Toronto, Ontario, M5S 3G4, Canada

^2^Department of Material Science & Engineering, University of Toronto, Toronto, Ontario, M5S 3E4, Canada

^†^Lawrence Berkeley National Laboratory, 1 Cyclotron Rd., Berkeley, CA, 94720, USA

[*Email: kherani@ecf.utoronto.ca](mailto:*Email:%20kherani@ecf.utoronto.ca), <http://www.ecf.utoronto.ca/~kherani/>

In this SI (supplementary information) of the article, we further investigate the conditions under which a width-graded grating structure obtains adiabatic mode transformation and thus enhanced multiwavelength light localization inside the grooves. We analyze the effect of adiabaticity in a width-graded grating structure by further looking at the surface component of the scattered electric field ($\vec{E}_{x}$), both its real part and phase, at groove-to-groove separation values of *d* = 300 nm and *d* = 100 nm. In this SI, we provide further justification of the results of Table 1, Figure 5, and Figure 6 presented in the article wherein we discussed multiwavelength light localization resulting from the strength of the SPPs propagating on the metallic surface of the grating and their coupling to the cavity modes, and also resulting due to adiabatic mode transformation among the grooves of a graded grating. The latter occurs when each of the grooves of a graded grating structure resonate strongly at a single wavelength while transferring higher order modes to adjacent groove(s).

Our results in Figure 5.b of the article revealed that at *d* = 300 nm improved phase engineering of SPPs leads to adiabatic mode transformation between adjacent grooves which in turn dictates the scattered electric field ($\vec{E}_{x}$) of all the other grooves, namely, pointing toward the resonating groove, or alternatively being zero. Figure S1.a displays the real part of $\vec{E}_{x}$ of a graded grating structure for *L* = 100 nm, *w* =10 nm, *Δ* = 5 nm, -4 ≤ *j* ≤ 0, and *d* = 300 nm. In Figure 6 of the article, the vectors pointing to the right (left) direction correspond to positive (negative) values of real part of $\vec{E}_{x}$ (Re($\vec{E}_{x}$)). Figure S1.b shows phase of $\vec{E}_{x}$ within different nano-groove widths across the visible and near-IR spectra. Within each groove, the phase has a positive (negative) value below(above) the resonance wavelength. At *d* = 300 nm, better adiabatic mode transformation is attained by virtue of dictating the $Re(\vec{E}_{x})$ in non-resonating grooves to point toward the resonating groove, and thus transferring higher order modes of each groove to the resonating groove. This effect mandates $Re(\vec{E}_{x})$ and phase of $\vec{E}_{x}$ in each groove to change sign only once around the resonant frequency of the groove, as shown in Figure S1.a and b where $Re(\vec{E}_{x})$ and phase of $\vec{E}_{x}$ of each groove crosses the value of zero only once in the vicinity of the groove’s resonant frequency.


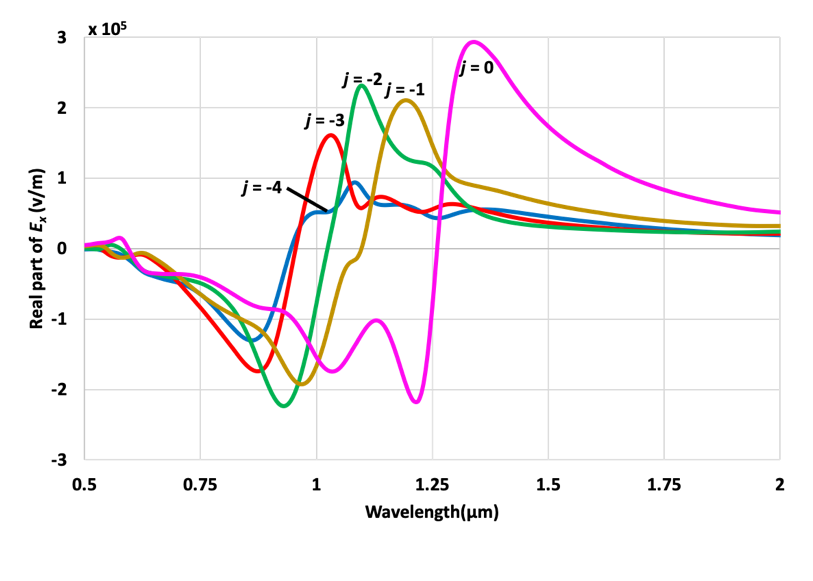


**Figure S1: a)** Real part of ${\vec{\boldsymbol{E}}}_{\boldsymbol{x}}$ and **b)** Phase of ${\vec{\boldsymbol{E}}}_{\boldsymbol{x}}$ values for a graded grating with L = 100 nm, w =10 nm, Δ = 5 nm, -4 ≤ j ≤ 0, and d = 300 nm. Note that Re**(**${\vec{\boldsymbol{E}}}_{\boldsymbol{x}}\boldsymbol{)}$ and phase of ${\vec{\boldsymbol{E}}}_{\boldsymbol{x}}$ cross the zero line only once for all grooves which indicates non-resonating grooves guide their higher order modes to their adjacent grooves thus improving adiabatic mode localization**.**


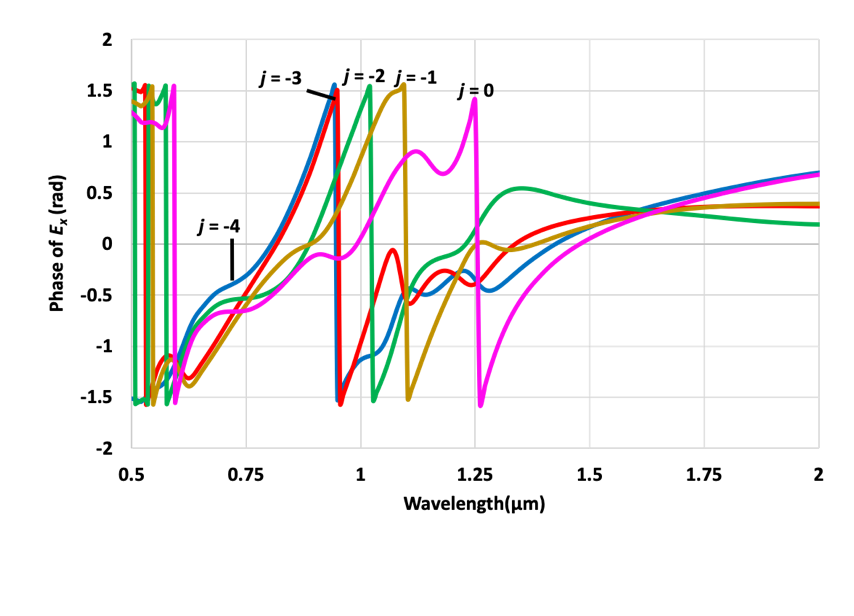


a

b

Figure S2.a displays Re($\vec{E}_{x}$) and Figure S2.b shows the phase of $\vec{E}_{x}$ within nano-grooves of a graded grating structure for *L* = 100 nm, *w* =10 nm, *Δ* = 5 nm, -4 ≤ *j* ≤ 0, and *d* = 100 nm. Note that in Figure S2 Re($\vec{E}_{x}$) and phase of $\vec{E}_{x}$ inside grooves *j* = -2 and *j* = -3 cross the zero value twice in the vicinity of the resonance wavelengths, *λ*_resonant_ = 1 µm and 1.06 µm, respectively. Thus, these grooves transfer non-resonant frequency modes to both narrower and wider neighboring grooves, and not only to the resonating groove as was the case for *d* = 300 nm where adiabatic mode transformation was in effect. Such multiple changes of direction of $\vec{E}_{x}$ on the surface of a graded grating at *d* = 100 nm does not lead to adiabatic mode transfer among the grooves and thus impairs multiwavelength light localization.

Visualization 1 shows changes in the strength and direction of $\vec{E}_{x}$ in two different structures of Figure S1 and Figure S2 for a spectral range of λ = 214 nm to λ = 600 nm.

Figure S3 presents the localized electric field profile for the same geometrical parameters as in Figure S2 where non-optimal phase engineering has led to an overlap of the resonant profiles of the grooves, with impaired multiwavelength light localization.


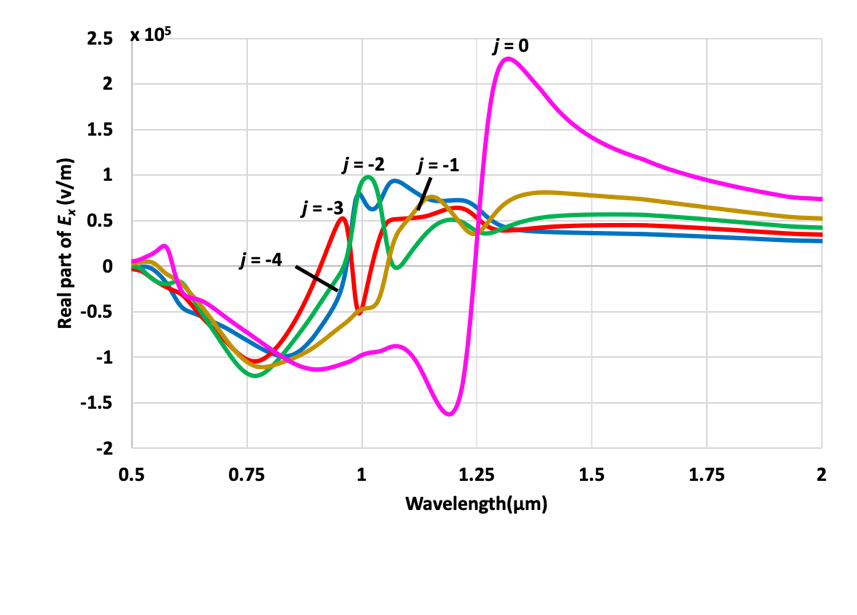


**Figure S2: a)** Real part of ${\vec{\boldsymbol{E}}}_{\boldsymbol{x}}$ **b)** phase of ${\vec{\boldsymbol{E}}}_{\boldsymbol{x}}$ values for a graded grating with L = 100 nm, w =10 nm, Δ = 5 nm, -4 ≤ j ≤ 0, and d = 100 nm, where values of Re**(**${\vec{\boldsymbol{E}}}_{\boldsymbol{x}}\boldsymbol{)}$ and phase of ${\vec{\boldsymbol{E}}}_{\boldsymbol{x}}$ cross the zero line two times for grooves j = -2 and j = -3 implying multiple changes on the direction of the surface component of the electric field on the surface of the grating which in turn impairs adiabatic mode transformation between the adjacent grooves.


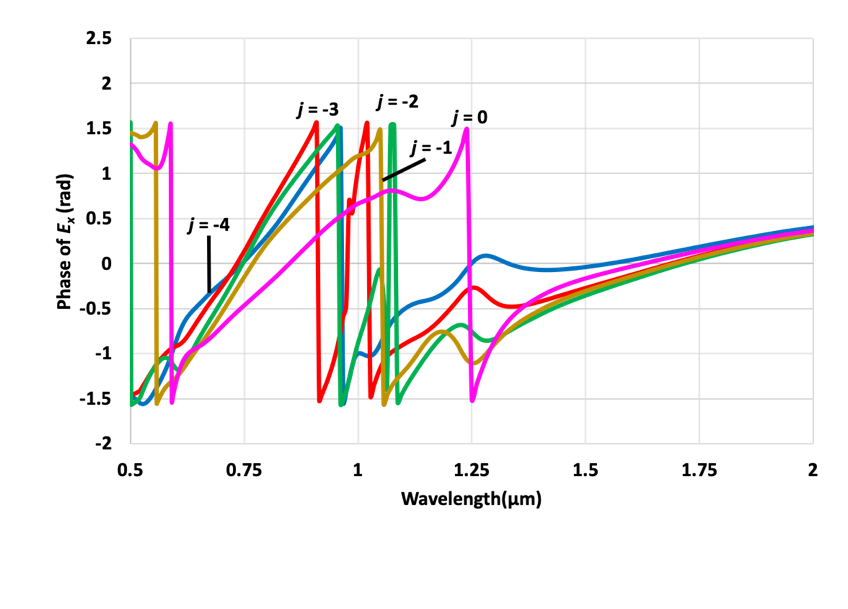


a

b


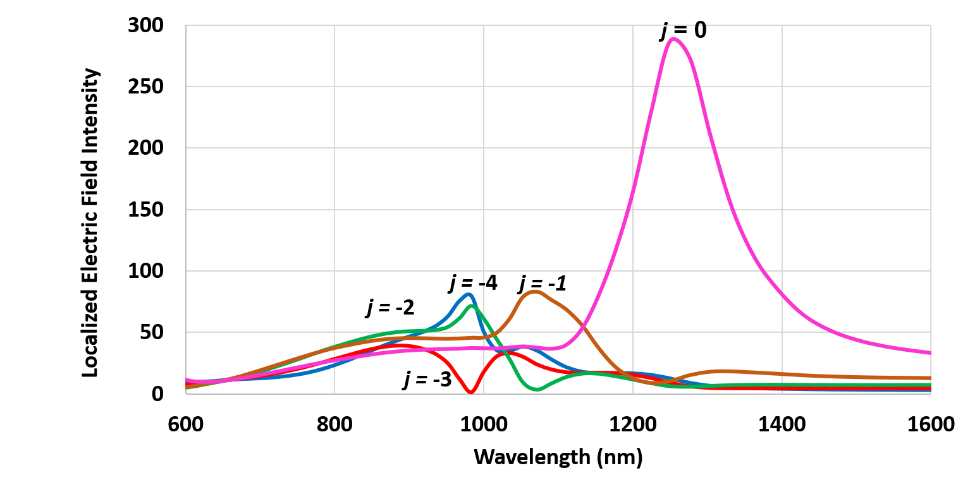


**Figure S3:** Localized electric field intensity for a graded grating with L = 100 nm, w = 10 nm, Δ = 5 nm, -4 ≤ j ≤ 0, and d = 100 nm. Multiwavelength mode localization in the graded grating with d = 100 nm is deteriorate due to a poor adiabatic mode transformation.

The localized electric field profile for graded grating structures with *d* < 50 nm and *d* > 900 nm are formed quite similarly, despite the different coupling mechanisms of light and SPPs leading to localization of electric field inside the grooves. Figure S4 and Figure S5 show the localized electric field intensity profiles for *d* = 30 nm and *d* = 1200 nm, respectively. In both cases, the narrowest groove (*j* = 0) localizes a moderately higher electric field compared to the wider neighboring grooves. It was elaborated in the article that, for *d* < δ_metal_ the evanescent mode-coupling between the adjacent grooves plays a significant role in defining the resonant profile of the structure. Figure S4 shows the impact of this evanescent mode-coupling on the overlapping resonant profile of different grooves and thus degradation of multiwavelength light localization in the structure. For *d* > 900 nm, SPPs propagate farther on the metallic surface of the grating prior to reaching the next groove and consequently they decay in intensity due to ohmic losses. Effect of this latter phenomena on the localized electric field profile of the structure is shown in Figure S5 where resonant peaks of the grooves start to overlap.


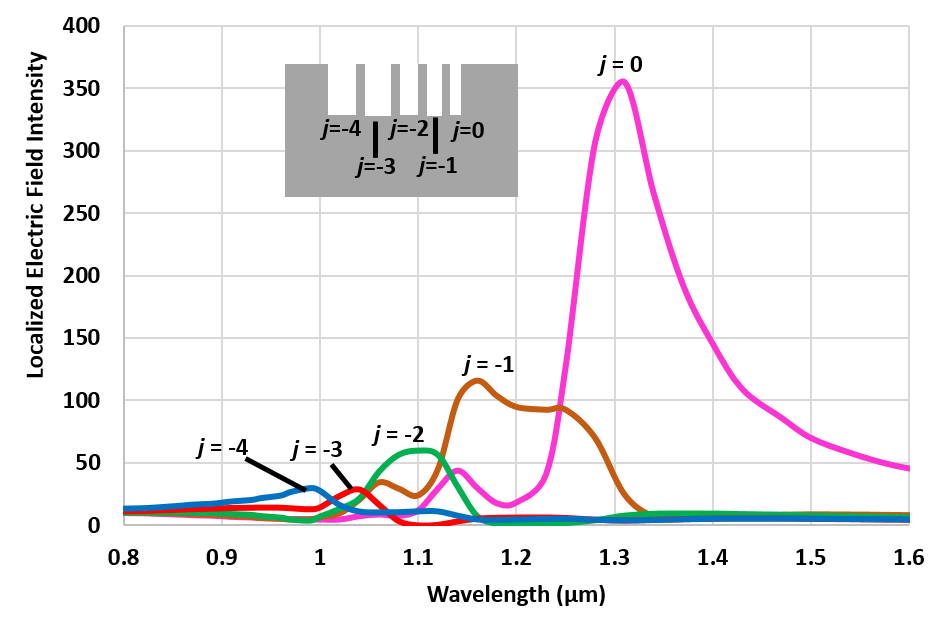


**Figure S4:** Localized electric field intensity for a graded grating with *L* = 100 nm, *w* = 10 nm, *Δ* = 5 nm, -4 ≤ *j* ≤ 0, and *d* = 30 nm; in this case *d* < δ_metal_, evanescent mode localization between the adjacent grooves defines the resonant profile of the structure and hence this localization mechanism impairs multiwavelength light localization inside the grooves

In both the structures of Figure S4 and Figure S5, strength of SPPs propagating on the grating surface are either poor or inconsequential to coupling to the cavity modes. This resonant behavior of the graded gratings, as was also presented in Table 1 of the article for a uniform lamellar grating, justifies that enhanced multiwavelength localization in a grating structure is attributed to the strength of the SPPs propagating on the grating surface and their coupling to the cavity modes where available.

Figure S6 shows the normalized field intensity map of a uniform width nano-grating for *L* = 500 nm, *w* =10 nm, *n* = 9 and -4 ≤ *j* ≤ +4, at a resonance wavelength of *λ* = 5.1 μm. This field intensity map corresponds to the first order resonant mode of the profile shown in Figure 5.a of the manuscript. Evidently, the localized electromagnetic field is distributed equally within all nano-grooves of the uniform width grating.


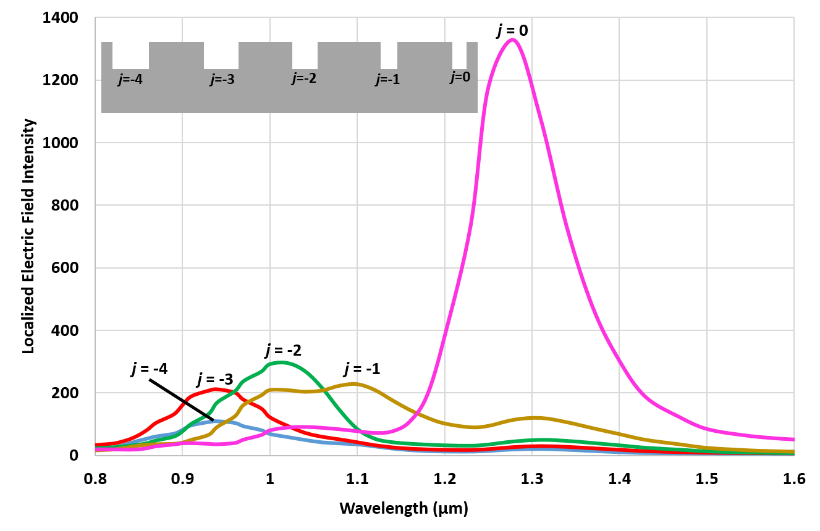


**Figure S5:** Localized electric field intensity for a graded grating with L = 100 nm, w = 10 nm, Δ = 5 nm, -4 ≤ j ≤ 0and d = 1200 nm: compared to smaller values of d, in this case SPPs propagate farther on the metallic surface of the grating prior to reaching the next groove and thus they decay due to ohmic losses. This phenomenon impairs hyperspectral mode localization inside the grooves.


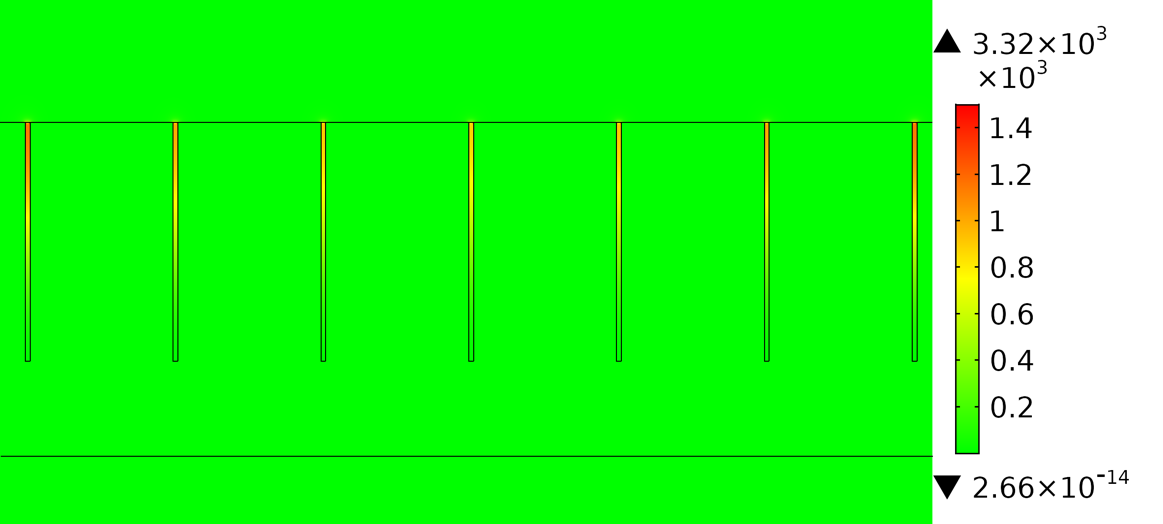


**Figure S6:** Localized electric field intensity for a uniform width grating with L = 500 nm, w =10 nm, n = 9 and -4 ≤ j ≤ +4: compared to smaller values of d, in this case SPPs propagate farther on the metallic surface of the grating prior to reaching the next groove and thus they decay due to ohmic losses. This phenomenon impairs hyperspectral mode localization inside the grooves.

The maximum *Q*-factor of the MIM width-graded nano-grating is calculated to be 20 for the 5th resonant mode at a groove depth of 500 nm and excitation wavelength of 680 nm (as shown in Table 2). Although, the *Q*-factors appear to be small, *Q*-factor values normalized to the volume (*Q/V*) for such subwavelength gratings are comparable to those of conventional optical resonators. Our width-graded nano-gratings have a minimum groove width of 10 nm, groove depth of 500 nm, and a groove length of at least 1µm. Hence, the minimum volume for a nano-groove is 5×10-21 (m3), giving a *Q*-factor per volume value of 4×1021 (m-3). Considering the modal volume for our nano-grating results in an even larger value for *Q/V*. However, here we consider the physical volume for a fair comparison with the optical resonators.

In contrast, optical resonators such as whispering-gallery modes resonators [1] or microsphere resonators [2] have a *Q*-factor of the order of 109-1010. Table S.1 lists two optical resonators with relatively high *Q*-factors along with their volumes and the values of Q-factor per volume. Whispering-gallery mode (WGM) resonator is a solid-state cavity capable of confining light in a micro-scale cavity. A quartz WGM resonator with a cavity width of 3.25 mm, length of 0.1 mm, and a depth of 220 µm (total volume of about 7×10-11 (m3)) was designed to have a *Q*-factor of 5×109 at a wavelength of 1550 nm. The *Q*-factor per unit volume for this optical resonator is about 7×1021 (m-3) [166]. In another study, Erbium-doped ZBLALiP micro-spheres were created with diameters between 50 µm to 150 µm. These have volumes ranging between 6.5×10-13 m3 to 1.76×10-12 m3; the *Q*-factor achieved for this micro-sphere is 5.294×109 at a wavelength of 1480 nm [167], hence the Q-factor per volume is 8.1×1023 (m-3).

**Table S1: Comparison of volume normalized quality factors, *Q*-factor per unit volume, for typical optical resonators and a width-graded nano-grating resonator**

| **Structure** | ***Q*-factor** | **Volume (m^3^)** | ***Q/V* ( m^-3^)** |
| --- | --- | --- | --- |
| Whispering-galley mode [1] | 5×10^9^ | 7.1×10^-11^ | 7×10^21^ |
| Microsphere resonator [2] | 5.294×10^9^ | 6.5×10^-13^ | 8.1×10^23^ |
| Width-graded Nano-grating | 20 | 5×10^-21^ | 4×10^21^ |

**Proof of the formula for the adiabatic parameter stated in Eq. (10):**

The adiabatic condition necessitates [3]:

$\left| \frac{\hbar\sigma^{''}(x)}{\left( \sigma^{'}(x) \right)^{2}} \right|\ll1$ (S1)

where $\hbar$ is the reduced Planck’s constant, $\sigma^{'}\left( x \right)$ and $\sigma^{''}(x)$ are the first and second derivatives of the $\sigma(x)$, the phase in the solution of Schrödinger’s equation wave function approximated by:

$\sigma(x)$≈$\int p\left( x \right)dx$, (S2)

where $p\left( x \right)$ is the momentum of a particle with a mass of $M$, total energy *E*, and potential energy *V*(x).

$\sigma'(x)$≈$\frac{d}{dx}\int p\left( x \right)dx=p(x)$ (S3)

$\sigma''(x)$≈$\frac{d^{2}}{dx^{2}}\int p\left( x \right)dx= \frac{d}{dx}p(x)$ (S4)

Given the momentum of a particle is defined as $p\left( x \right)=\sqrt{2M\left[ E-V(x) \right]}= \hbar\beta$ [3], and following Eqs. (S3) and (S4):

$\sigma^{'}=\hbar\beta$ (S5)

$\sigma^{''}=\hbar\frac{d\beta}{dx}$ (S6)

Substituting Eq.(S6) into Eq. (S1):

$\left| \hbar\frac{\hbar\frac{d\beta}{dx}}{\left( \hbar\beta\right)^{2}} \right|=\left| \frac{d\beta}{dx}\frac{1}{\beta^{2}} \right|=\left| \frac{d\beta^{-1}}{dx} \right|$ (S7)

Eq.(S7) may be rewritten as:

$\delta\cong\frac{\frac{1}{\beta_{1}}-\frac{1}{\beta_{2}}}{\Delta x}$ (S8)

In relationship to the groove indices, Eq.(S8) can be written as follows which is Eq.(10) in the manuscript:

$\delta_{j}=\frac{\frac{1}{\beta_{j}}-\frac{1}{\beta_{j+1}}}{Ʌ_{j}}$ (S9)

**References:**

[1] Iichenko, A. A. Savchenkov, J. Byrd, I. Solomatine, A. B. Matsko, D. Seidel, and L. Maleki, “Crystal
 Quartz Optical Whispering-gallery Resonators”, Optics Letter, Vol. 33, No. 14, 2008.

[2] R. Henriet, P. Salzenstein, D. Ristic, A. Coillet, M. Mortier, A. Rasolaniaina, K. Saleh, G. Cibiel, Y.
 Dumeige, M. Ferrari, Y. K. Chembo, O. Liopis, and P. Feron, “High Quality-Factor Optical
 Resonators”, Physica Scripta, 2014.

[3] L. D. Landau and E. M. Lifshitz, Quantum mechanics - Non-relativistic theory, London: Pergamon
 Press, 1962.
